# Sextortion: A Scoping Review

**DOI:** 10.1177/15248380241277271

**Published:** 2024-09-25

**Authors:** Alana Ray, Nicola Henry

**Affiliations:** 1 RMIT University, Melbourne, Australia

**Keywords:** sextortion, sexual extortion, cyber blackmail, image-based sexual abuse, revenge porn, technology-facilitated sexual violence

## Abstract

Sextortion refers to making threats to share nude or sexual images to coerce the victim into complying with certain demands, such as paying a ransom, sharing intimate images, or engaging in unwanted acts. Sextortion occurs in a diverse range of contexts, including intimate partner abuse, cyberbullying, sexuality or sex worker “outing,” online dating, “cybersex,” sex trafficking, online sexual exploitation of children, computer hacking, and organized crime. Despite the heightened media focus, few studies have measured the prevalence, nature, and impacts of sextortion. We conducted a scoping review with the aim of providing a comprehensive overview of existing empirical research on sextortion victimization and perpetration among youth and adults. In total, 24 studies were identified based on predefined eligibility criteria. We found that studies focusing on youth reported prevalence ranging from 0.7% to 5.0%, while studies involving adults ranged from 4.0% to 18.7%. The review found that young people and sexual minorities are more likely to experience victimization, while men, young people, and sexual minorities are more likely to self-report engaging in sextortion offending. The review found that perpetrators are more likely to be intimate partners or other known persons as opposed to strangers and that there was an overlap between sextortion perpetration and victimization. Finally, we also found that sextortion can result in significant harms, and that reporting and help-seeking remain very low due to shame, fear, and negative perceptions of police and digital platforms. The findings highlight existing gaps and provide recommendations for future research, policy, and practice.

## Introduction

Sexual extortion (“sextortion”) is a prevalent and pernicious form of coercion that refers to making threats to share nude or sexual images to coerce the victim into complying with certain behavioral or financial demands. These demands may include monetary payment, intimate images, engagement in sexual acts, or compliance as part of a pattern of power and control. “Intimate images” refers to photos or videos of a person’s genital, anal, or breast region, or of a person or persons engaged in a sexual act. Sextortion is both a form of image-based sexual abuse (IBSA) (Henry et al., 2020)^1^ and technology-facilitated sexual violence (TFSV) involving digital technologies in communicating the threat (Powell & Henry, 2017).^2^ In addition, sextortion can also be a form of gender-based violence because not only does sextortion occur in the context of intimate partner abuse but also intimate images have significant potency due to the potential shame and stigma attached to a person’s gender and sexuality, as well as other markers of identity, such as race, age, and ability.

Sextortion can occur in a variety of contexts and is perpetrated by a diverse group of offenders, both known and unknown to the victim. For example, sextortion can be perpetrated by abusive intimate partners who threaten to share intimate images to prevent the victim from leaving the relationship, taking custody or parental responsibility of children, pursuing an intervention order, or as another form of power and “coercive control” (Henry et al., 2023; Vitis, 2020). Sextortion can be perpetrated as part of organized crime, whereby the offender pretends to be someone else online and then lures the victim into sharing intimate images and blackmails them for financial gain (Cross et al., 2022; Foster, 2023). Other instances of sextortion involve sex traffickers who threaten to send intimate images to the victim’s family members in their country of origin to obtain compliance (Gezinski & Gonzalez-Pons, 2022; Perkins & Ruiz, 2017); sexual predators who groom or coerce young people into sharing their intimate images (Finkelhor et al., 2023); people engaged in online dating or online sex who threaten to share intimate images as a way to “out” or shame the victim or have some control over them; scammers who claim they have recorded the victim visiting adult websites; or computer hackers who trick victims into downloading malware to enable access to their intimate images through their personal files or webcams (O’Malley & Holt, 2022; Wittes et al., 2016).

Research has shown that the impacts and harms of sextortion can be significant and life-changing. Victims of sextortion often experience fear, humiliation, shame, and self-blame (Henry et al., 2023; Mandau, 2021; Trendell, 2019; Walsh & Tener, 2022), which can manifest as depression, anxiety, suicidal ideation, and self-harm (Gámez-Guadix, Mateos-Pérez et al., 2022; Walsh & Tener, 2022; Wolak et al., 2018). Victims of sextortion can also be impacted financially when they comply with monetary demands, as well as socially, when they experience a deterioration in relationships with family and friends or change their jobs, career pathways, or schools as a result (Walsh & Tener, 2022; Wolak et al., 2018).

Recently, there has been an increase in media addressing the issue of sextortion, as law enforcement and online safety agencies report significant increases in the number of people reporting sextortion victimization, particularly in the context of financial scams (e.g., Lybrand, 2022; Richards, 2023). By the end of 2022, for instance, the FBI reported a tenfold increase in sextortion reports (Whitehurst, 2022). A study focusing on victim reports of cybercrime reported to BitcoinAbuse found that sextortion was the second most reported crime after blackmail (Buil-Gil & Saldaña-Taboada, 2022). In 2023, New Zealand’s online safety agency, Netsafe, announced an 88% increase in the reports of sextortion since 2019 (Owen, 2023). Sextortion is also currently the most reported issue to the U.K. Revenge Porn Helpline (Crew, 2022) and the most reported form of IBSA according to the Australian eSafety Commissioner’s latest annual report, with most victims being young men between 18 and 24 years (Australian Communications and Media Authority and Australian Office of the eSafety Commissioner, 2024).

While the figures from online safety and law enforcement agencies demonstrate that sextortion is a growing problem and that young men are more susceptible, there is a surprising lack of empirical research on the topic. Moreover, the data from law enforcement or online safety agencies are likely to be an underestimate of the true prevalence of sextortion. Research shows that many victims under-report their experiences due to factors such as shame, a lack of evidence, difficulty identifying the perpetrator, fear of not being taken seriously by authorities, or a desire to protect a perpetrator known to them, such as an intimate partner (Henry & Umbach, 2024; Patchin & Hinduja, 2020; Wolak et al., 2016).

Understanding the prevalence, nature, and impacts of sextortion, including how gender, age, race/ethnicity, and sexuality influences perpetration and victimization, is crucial for shaping laws, policies, and intervention strategies to more effectively detect, prevent, and respond to this growing problem. This study provides an overview of the existing research on sextortion using a scoping review methodology. A scoping review is useful for mapping the available literature on a given topic, particularly when there is a dearth of literature (Hanneke et al., 2017; Munn et al., 2018). To our knowledge, this is the first scoping review on sextortion to be undertaken to date. In this article, we report on key findings from the empirical literature on sextortion in relation to prevalence, impacts, and help-seeking behaviors among youth and adults. We also identify gaps and make several recommendations for future research, policy, and practice.

## Research Design and Methodology

A scoping review was undertaken to develop a comprehensive understanding of the prevalence, nature, and impacts of sextortion. The scoping review followed the guidelines outlined in the Preferred Reporting Items for Systematic Reviews and Meta-Analyses Extension (PRISMA-ScR) methodology (Munn et al., 2018; Tricco et al., 2016). We closely followed the procedures and protocols for conducting scoping reviews, including: formulating clearly defined research questions; using a tried and tested search strategy; adopting a transparent and consistent study selection strategy driven by agreed-upon inclusion and exclusion criteria; presenting and analyzing study findings; and discussing implications for future research, policy, and practice. The research questions guiding the study were as follows:

1. What is the prevalence of sextortion, and what are the demographic risk characteristics?
2. What are the impacts or harms of sextortion?
3. What are the barriers to reporting and/or disclosing experiences of sextortion?

### Search Strategy and Study Selection

In August 2023, we conducted a comprehensive search of the empirical literature on sextortion across four databases: SCOPUS, ProQuest, PsycInfo, and Web of Science. We also searched for additional articles in Google and Google Scholar and reviewed the reference lists of key studies on sextortion. We used a comprehensive list of search terms based on our existing knowledge of the field (see Table 1). These search terms were thoroughly tried and tested across the four databases over multiple meetings. We chose to focus on sextortion as a form of IBSA and, therefore, restricted our search to “threats to share intimate images” rather than also including threats to share other content, such as audio recordings, text messages, or evidence of someone visiting a pornographic site. Our definition of “intimate images” included nude or sexual images; therefore, we did not search for “embarrassing” or other sensitive images (e.g., a photo or video of someone intoxicated at a party).

**Table 1.** Keywords for Database Searches.

Sextortion Terms	Blackmail and Extortion Terms	Image-based Abuse Terms
“sextortion” OR “sexual extortion” OR “sexual blackmail” OR “intimate blackmail” OR “cyber blackmail” OR “online blackmail” OR “sex scam” OR “digital blackmail” OR “cyber exploitation” OR “sexual scam” OR “photo blackmail” OR “nude scam” OR “digital extortion” OR “online extortion” OR “cyber extortion”	extort* OR threat* OR blackmail* OR scam*	“image-based sexual abuse” OR “image-based abuse” OR “revenge porn” OR “revenge pornography” OR “non-consensual porn” OR “nonconsensual porn” OR “non-consensual pornography” OR “nonconsensual pornography” OR “intimate image” OR “sexual image” OR “nude image” OR “explicit image” OR “private image” OR “explicit video” OR “nude video” OR “intimate video” OR “explicit picture” OR “nude photo” OR “sexual photo” OR “explicit recording” OR “nude picture” OR “sexual video” OR “sexual picture” OR “private video” OR “intimate recording” OR “sexual recording” OR “private recording” OR “private picture” OR “explicit photo” OR “private photo” OR “intimate picture” OR “intimate photo” OR “nude recording”

We (the two authors) agreed on inclusion and exclusion criteria prior to undertaking the search for studies. Studies were included if they (a) reported on the findings of scholarly or gray empirical (quantitative or qualitative) research on sextortion where study participants were either victims or perpetrators; (b) were published between 2013 and 2023; (c) were published in English; and (d) reported on findings in relation to sextortion perpetration or victimization where threats had been made to disseminate nude or sexual images. In addition to excluding nonempirical studies, non-English studies, and those published before 2013, we excluded studies solely reporting on qualitative interviews with professional organizations (e.g., Caarten et al., 2022) or interviews with only a single-victim survivor (e.g., Howard, 2019). Furthermore, we excluded studies that treated sextortion in much broader terms, for instance, within the workplace where a person is compelled into sexual activity to get a job, a promotion, or to obtain money or other resources (e.g., Mumporeze et al., 2021). We also excluded studies on “sexting coercion,” which refers to someone pressuring, coercing, or threatening another person into sharing their intimate images (e.g., Gassó et al., 2021). Sexting coercion is distinct from sextortion, which, for the purposes of this review, we defined broadly as *making threats to share intimate images.*

The bibliographic details of the studies identified through the four database searches (*n* = 938), Google Scholar references (*n* = 91), and other searches (e.g., Google search; references list of key articles) (*n* = 9) were exported into a Microsoft Excel spreadsheet, yielding a total of 1,038 unique entries. Duplicate studies and other irrelevant sources (e.g., non-English sources, errata, and editorials) were removed (*n* = 494). We then independently reviewed the title and abstracts of the remaining sources (*n* = 544) to ensure consistency in screening and the application of the inclusion criteria. We each used the highlighter tool in Microsoft Excel to independently color-code the sources in the spreadsheets, with different colors indicating their potential alignment with our inclusion criteria: yellow for articles that appeared to meet the criteria, blue for articles where there was uncertainty, and green for articles that did not meet the criteria. This enabled us to compare selections as well as review any disagreements and resolve any inconsistencies through multiple meetings.

**Figure 1.**
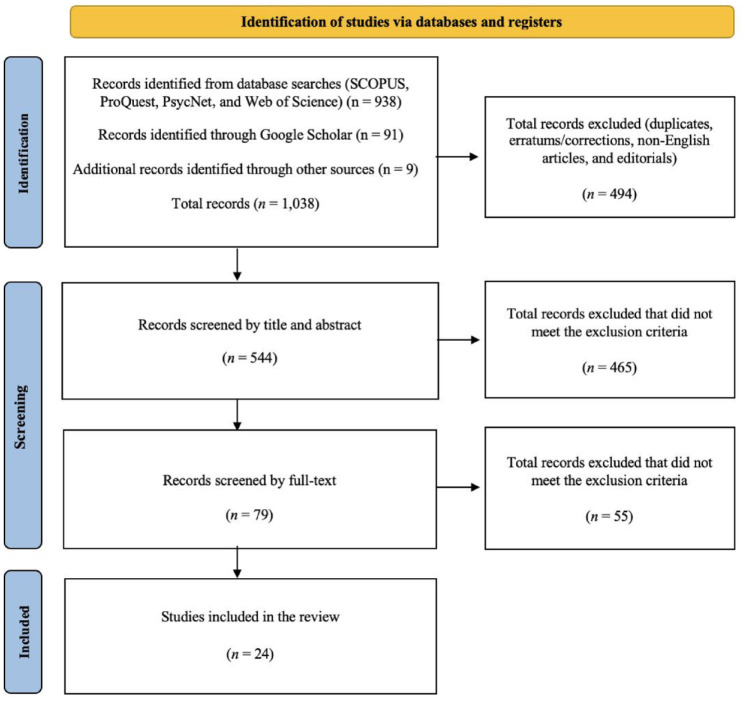
Identification of studies via databases and registers using the Preferred Reporting Items for Systematic Reviews and Meta-Analyses Extension framework.

Through this process, we removed 465 duplicates or irrelevant articles, leaving a total of 79 eligible sources. In the final selection phase, we obtained the full texts of all 79 articles and saved them in a shared folder. The first author and a Research Assistant (who was not involved in the study design or abstract selection process) then conducted a full-text review of each study and entered details into the spreadsheet. The second author then reviewed the descriptions in the spreadsheet and checked the full text of all 79 articles to further determine which articles did not meet the criteria. A meeting was convened between the first and second authors to discuss the reasons for excluding the additional articles, leading to an agreement to remove a further 55 articles. This left a total of 24 articles to be included in the final review (see Figure 1).

### Charting and Collating the Data

The data were charted using a structured data extraction spreadsheet in Microsoft Excel to systematically capture the following information: (a) authors; (b) citation; (c) definition of sextortion (if included); (d) focus/aims; (e) research method (i.e., qualitative, quantitative, or mixed method); (f) description of the study (i.e., details regarding the study population and setting, whether it was representative, quota, or convenience, etc.); (g) relevant key findings (based on our research questions); and (h) limitations. Each of the 24 articles was carefully read again by the second author to extract the key findings of each study across five key categories: (a) terminology, (b) prevalence, (c) types of offending, (d) impacts and harms, and (e) reporting and help-seeking. The results of the study are discussed below.

## Results

The 24 articles included in this scoping review span publication dates from 2016 to 2023, with 14 published between 2020 and 2023 (58.3%). The studies were conducted in the following countries: the United States (*n* = 8), Spain (*n* = 4), Australia (*n* = 3), Canada (*n* = 2), Czech Republic (*n* = 1), Denmark (*n* = 1), New Zealand (*n* = 1), Singapore (*n* = 1), and Switzerland (*n* = 1). Additionally, two cross-country studies were conducted in Australia, New Zealand, and the United Kingdom (*n* = 2). Most studies (*n* = 17, 70.8%) reported on quantitative research, with 16 studies conducting surveys and one analyzing sextortion spam emails. The sample sizes in the quantitative survey studies ranged from 630 to 6,109. The remaining studies (*n* = 6, 25.0%) reported on qualitative research, including two studies involving interviews with victim-survivors, two analyzing reports made to authorities, and two analyzing social media posts from victim-survivors of sextortion. Finally, one study (*n* = 1, 4.2%) adopted a mixed-methods approach to examine victim-survivor reports of cyber abuse. Results are presented below (see also Table 2).

**Table 2.** Summary of Studies Included in the Review.

Study	Study Design	Sample	Focus/Aim	Key Findings
Buil-Gil and Saldaña-Taboada (2022)	Mixed-methods (quantitative and qualitative analysis of victim reports of cyber abuse)	(*n* = 186,735) United States	To uncover the concentration of cybercrime offending and identify groups of offenders engaged in online crime.	Sextortion was the second most reported crime, trailing only behind blackmail. The nature of threats included blackmail for sexual favors or as a means to prevent the dissemination of explicit information, with offenders demanding ransom. Reporting occurred through the “BitcoinAbuse” website.
Cross et al. (2022)	Qualitative thematic analysis of cases of romance fraud reported to Scamwatch (Australia) and typology analysis	(*n* = 258) Australia; Adults (23.0% women, 76.0% men, 1.0% unspecified gender)	To explore the use of sextortion within the context of romance fraud.	Sextortion, affecting both men and women, primarily occurs within existing relationships, and often escalates within the context of romance fraud. Incidents of sextortion lead to significant financial and nonfinancial harms for the victims, who reported these incidences through platforms like Scamwatch and the Australian Competition and Consumer Commission.
Canadian Center for Child Protection (2022)	Qualitative thematic analysis of Reddit r/Sextortion forum.	(*n* = 478) Canada (98% male; 2.0% female)	To investigate the prevalence and characteristics of financial sextortion as reported on the r/Sextortion forum on Reddit.	Findings reveal that boys and young men were the primary targets of financial sextortion. Perpetrators commonly employ the strategy of posing as females on Instagram to initiate contact before transitioning conversations to Snapchat where they coax victims into sharing nude images. These images are then used to blackmail victims for money, with threats to distribute them to the victim’s social circles. Compliance with sextortion demands often leads to escalating requests for funds. Victims often express disappointment when seeking help from cybersecurity or reputation management firms, and they also expressed frustration with the inadequate responses they received.
Eaton et al. (2022)	Survey	(*n* = 2,006) United States; Adults (18+) (52.4% women)	To explore the relationship between pre-COVID-19 intimate partner violence (IPV) victimization and sextortion victimization during the COVID-19 pandemic.	Pre-pandemic sexual IPV was a predictor of sextortion, particularly among those aged 18–29. Men, Native American, Black women, and sexual identity minorities reported higher victimization rates. Perpetrators included strangers, romantic partners, friends, and online acquaintances. Men were more frequently victimized than women during the pandemic.
Finkelhor et al. (2022) (same study also reported in Finkelhor et al. 2023)	Survey	(*n* = 2,639) United States; Adults (18–28) (49.8% women; 49.8% men; 1.8% other gender)	To analyze the characteristics of online sexual abuse and cyberstalking episodes and their association with negative emotional impacts.	Respondents were adults reporting experiences both before and after turning 18. Women were the primary targets of sextortion when they were under 18, with most perpetrators being men. Adults were less likely to be offenders than youth. Intimate partners were more likely to be perpetrators, engaging in various online offenses, such as nonconsensual image sharing and cyberstalking, with adults being more involved in specific activities. Most incidents resulted in negative emotional impacts, especially those involving nonconsensual image sharing and sextortion.
Gámez-Guadix and Incera (2021)	Survey	(*n* = 1,779) Spain; Adolescents (12–18) (50.9% girls)	To analyze whether online sexual victimization and risks mediate the relationship between being a sexual minority and mental health outcomes, specifically depression and anxiety.	Sexual minority adolescents reported a higher prevalence of sextortion compared to their heterosexual counterparts. Interestingly, sextortion did not show a significant impact on mental health outcomes, in contrast to the patterns observed with other forms of online sexual victimization.
Gámez-Guadix, Mateos-Pérez et al. (2022)	Survey	(*n* = 1,820) Spain; Adolescents (12–17) (51.0% girls; 48.2% boys; 0.16% nonbinary; 0.6% no gender specified)	To analyze the prevalence of sextortion and nonconsensual sexting.	Sextortion perpetration is less common in comparison with nonconsensual sexting perpetration. Using the Image-Based Sexual Abuse (IBSA) Scales, the research identified a strong correlation between sextortion and nonconsensual sexting, with stability patterns varying based on gender. Both forms of IBSA were associated with symptoms of depression and anxiety, emphasizing the need for early education on sexting.
Gámez-Guadix, Sorrel et al. (2022)	Survey	(*n* = 1,682) Spain; Adolescents (12–18) (51.4% girls; 48.5% boys; 0.1% no gender specified)	To analyze the relationship between different forms of technology-facilitated sexual violence (TFSV) perpetration and victimization.	Distinct victimization profiles and interconnectedness in various forms of TFSV were observed. Girls generally reported higher scores for sexual victimization variables, but no significant gender differences were found in sextortion. The study also emphasized the need for preventive programs addressing different TFSV types and interventions that consider prior victimization history among aggressors. Gender differences in victimization and perpetration highlight the importance of routinely including TFSV in offline harassment prevention campaigns.
Henry et al. (2019) (same study also reported in Powell et al., 2019)	Survey	(*n* = 4,274) Australia; Adults (16–49) (56% women; 44% men)	The study aimed to collect empirical data on the nature, prevalence, and impacts of IBSA.	About a quarter of survey participants reported experiencing some form of IBSA victimization. The most common forms were the nonconsensual taking and distribution of intimate images. Men and young people (18–29) were more likely to report sextortion perpetration behaviors. Higher rates of sextortion victimization were observed among young people, LGBTQI+ individuals, those with disabilities, and Aboriginal and Torres Strait Islander people.
Henry et al. (2020) (same study also reported in Powell et al., 2022a, 2022b)	Survey	(*n* = 6,109) Australia, New Zealand, United Kingdom; Adults (16–64) (52.1% women; 47.9% men)	The study aimed to investigate the prevalence, nature, and IBSA.	One in three respondents had experienced at least one form of IBSA victimization since the age of 16, including almost one in five who experienced someone threatening to share their nude or sexual image. The study also found that one in six had engaged in IBSA perpetration, including one in twelve who had threatened to share someone else’s nude or sexual images. Men, Indigenous people, Black, Asian, and Minority Ethnicity (BAME), lesbian, gay, and bisexual individuals, and young people were more likely to be both victims and perpetrators of sextortion.
Henry et al. (2023)	Qualitative interviews	(*n* = 30) Australia, New Zealand, United Kingdom; Adults (18+) (96.7% women; 3.3% gender-diverse)	To examine the experiences of individuals who have experienced IBSA as part of a pattern of coercive control.	IBSA manifested significantly in abusive relationships, where partners used intimate images for intimidation and degradation. Coercive control tactics, such as threats to share images, created fear and anxiety. This form of abuse led to emotional harm, loss of control, and various impacts on victims’ well-being. The challenges of reporting and seeking help were pronounced due to fear and entrapment.
Kopecký (2017)	Survey	(*n* = 21,372) Czech Republic; Children (11–17) (55.4% girls; 44.6% boys)	To assess the prevalence of extortion among Czech children and investigate the specific techniques employed by perpetrators to engage in blackmail.	This study found a rising trend in online blackmail cases among Czech minors. Offenders use comparable tactics to manipulate and exploit victims emotionally. Girls, particularly those aged 15–17, were more vulnerable to sextortion than boys. Child victims seldom reported incidents of blackmail to trusted adults.
Mandau (2021)	Qualitative thematic analysis of posts from an online counseling hotline (BørneTelefonen)	(*n* = 157) Denmark	To explore female adolescents’ experiences of IBSA victimization through a qualitative analysis of anonymous posts obtained from an online counseling hotline.	Snapchat serves as a common platform for IBSA. Victims often grapple with feelings of self-blame and psychological harm, experiencing a range of emotions such as worry, panic, and sadness. When it comes to reporting and seeking help, victims commonly resort to using blocking features on social media platforms.
Pacheco et al. (2019)	Survey	(*n* = 1,001) New Zealand; Adults (18+) (52% women; 47.7% men)	To investigate the prevalence of, and attitudes toward, IBSA.	A small percentage of participants reported having experienced threats to share intimate content online, with similar rates among women and men. Nonheterosexual individuals experienced slightly more threats than heterosexuals, and younger adults faced higher risks than older age groups. Threats were more common among Asian and Māori respondents. Ex-partners and strangers/unknown individuals were the primary perpetrators, with motives including jokes, seeking money, revenge, or gaining control. Only a small number admitted to threatening to share someone else’s intimate content in the past year.
Paquet-Clouston et al. (2019)	Quantitative analysis of sextortion spam emails	*(n* = 4,340,736) Switzerland; Spam emails	This research aimed to understand sextortion by uncovering spammers’ operations and demonstrating the prevalence of cryptocurrency, particularly Bitcoin, in sextortion cases.	Findings reveal a surge in sextortion cases involving Bitcoin payments, showcasing sophisticated strategies in pricing and the lucrative nature of spamming. A single entity was identified as likely overseeing the financial infrastructure of numerous sextortion campaigns, yielding substantial revenue.
Patchin and Hinduja (2020)	Survey	*(n* = 5,568) United States; Adolescents (12–17) (50.1% girls; 49.9% boys)	To explore the prevalence of sextortion behaviors among a nationally representative sample of U.S. middle and high school students.	Boys and nonheterosexual youth were more likely to be both victims and perpetrators of sextortion. Harms included stalking, harassment, fake profiles, and “revenge porn-like” incidents. The impact involved a lack of trust in adults, fear of retaliation, shame, and uncertainty about seeking help. Youth, regardless of gender, exhibited limited confidence in authorities, with few reporting incidents to relevant sites or apps.
Powell and Henry (2019)	Survey	(*n* = 2,956) Australia; Adults (18–54) (50.1% women; 49.1% men)	To investigate the prevalence, nature, and impacts of technology-faciltiated sexual violence (TFSV) victimization among Australian adults.	Findings revealed that over half of the respondents had experienced TFSV. A notable proportion of respondents reported receiving threats from perpetrators to share their personal information, such as intimate photos or private messages, if they did not comply with demands or requests. These threats exacerbate feelings of vulnerability and coercion among victims, highlighting the manipulative tactics employed by perpetrators.
Tamarit et al. (2021)	Survey	(*n* = 1,763) Spain; Adolescents (12–16) (51.0% girls; 49.0% boys)	To examine the interplay between adolescents’ addiction to social networks and the internet, body self-esteem, and sexual-erotic risk behavior online (including sexting, sextortion, and grooming).	Internet addiction, specifically “geek behavior,” was found to be a predictor of sextortion. The study identified two types: erotic, involving harmful content exchange, and coercive, focused on publishing explicit photos. Online victimization was linked to body self-esteem and sexting. While reporting specifics were not addressed, the study emphasized the importance of preparing adolescents for online harassment and promoting reporting to parents, teachers, and professionals.
Thorn (2017)	Survey	(*n* = 2,097) United States; Adolescents and adults (13–25) (91.0% women)	To provide an initial understanding of the characteristics of sextortion, its impact on victims, and strategies for enhancing the protection of children and young people from sextortion.	Sextortion significantly impacts young people, manifesting in threats and explicit demands across diverse platforms. It occurs in both new and existing relationships, whether online or offline. Victims often experience profound emotions of shame, embarrassment, and fear. Many choose not to report, believing they can handle the situation themselves. Reporting to friends or family members is more common than reporting to platforms or law enforcement, although responses from these entities are often inadequate.
Trendell (2019)	Survey	(*n* = 630) Canada; Adults (66.8% women; 30.0% men)	To comprehend the risk factors and consequences associated with inappropriate intimate image-based behaviors among young adults.	High rates of nonconsensual sharing and sextortion, particularly impacting women and individuals in gender or sexual minority groups, were uncovered in this study. The research explored the broader context of inappropriate intimate image-based behaviors, revealing correlations with increased mental health symptoms, regardless of prior victimization.
Vitis (2020)	Qualitative thematic analysis of image-based abuse reports made to a local sexual assault service provider	(*n* = 30) Singapore; Adults	To map the different ways image technologies impacted Singaporean women’s experiences of sexual violence.	Sextortion was recognized as a means of coercion, humiliation, and abuse, affecting different aspects of women’s lives and reinforcing gender-based power dynamics. Victims faced significant emotional distress and were reluctant to seek support. The study highlighted the role of threats in enforcing compliance.
Walsh and Tener (2022)	Qualitative interviews	(*n* = 48) United States; Adults (18–25) (73.0% women; 23.0% male; 2.0% unspecified)	To examine sextortion as an emerging type of victimization.	Instances of sextortion were characterized by brief, emotionally distant online encounters, with perpetrators often concealing their identities on platforms like Skype, MySpace, Messenger, Facebook, Snapchat, Whisper, Teenspot, Meet Me, and Grindr. Respondents detailed negative emotions such as anxiety and fear, along with attempts to negotiate, question, or emotionally appeal to perpetrators. Compliance with demands often led to escalating threats, such as image publication or further explicit image requests. Reasons for not disclosing were often due to feelings of shame, fear of judgment, and concerns about potential repercussions from family, peers, or law enforcement.
Wolak et al. (2018)	Survey	(*n* = 1,385) United States; Adults (18–25) (91.0% women; 9.0% men)	To educate the public and practitioners about sextortion, improve reporting mechanisms, to better equip technology companies with more knowledge and information, and to encourage help-seeking.	Sextortion manifested in both face-to-face and online relationships, involving threats, image dissemination, and other impacts such as stalking and assault. Reporting hurdles included the lack of specific laws, jurisdictional issues, and difficulties proving perpetrator identity, with minors facing shaming and threats of child sexual abuse material prosecution when seeking help from law enforcement.
Wolak et al. (2016)	Survey	(*n* = 1,631) United States; Adults (18–25) (83.0% women; 14.0% men)	To combat sextortion, educate the public, enhance tech platform reporting, provide knowledge to tech companies, and support targets in seeking help.	Sextortion incidents, particularly among minors, involve known perpetrators coercing victims into creating explicit images. Many victims, influenced by fear and embarrassment, choose not to disclose. The impact is profound, affecting relationships, mental health, education, and residence. The study also notes limited victim reporting.

### Terminology

There was consensus among the 24 studies we reviewed that sextortion involves three key components: first, some kind of *threat*; second, the threat must be to share *intimate (nude or sexual) images* with others; and third, the threat is accompanied by a *demand*, such as paying money, engaging in unwanted acts, or sharing intimate images. The 24 studies we identified were distinct from other studies on IBSA in the broader literature, including those on “sexting coercion” (which refers to pressure, coercion, or threats made against the victim to force them to share their intimate images) (e.g., Gassó et al., 2021), the nonconsensual sharing of intimate images (e.g., Ruvalcaba & Eaton, 2020), or sending unwanted sexually explicit images (“cyberflashing”) (e.g., Karasavva et al., 2023).

There were, however, some slight differences in how sextortion was defined or operationalized among the different studies. First, although the majority of studies defined or discussed sextortion as threats to share intimate (nude or sexual) images (photos or videos) (*n* = 22, 91.7%), two studies (8.3%) mentioned intimate images as well as intimate content or information, such as evidence of the victim visiting pornographic sites or content found on the victim’s computer (Buil-Gil & Saldaña-Taboada, 2022; Gámez-Guadix, Mateos-Pérez et al.. 2022). Second, there was a lack of consistency among the different studies regarding what the demand or ransom entails in the threat. For instance, one study defined sextortion as threats to share intimate images to demand *sexual favors or sex* (Wolak et al., 2018). In another study, it was *money* only (Paquet-Clouston et al., 2019), while a different study specified *sexual favors, money, and/or additional intimate images* (Finkelhor et al., 2023). The remainder of the studies implied or explicitly mentioned a broader range of demands, including money, sexual favors, and/or specific behavioral demands, such as sending more intimate images, staying in an intimate relationship, or other reasons, such as for revenge or humiliation. For example, Gámez-Guadix, Mateos-Pérez et al. (2022, p. 789) defined sextortion as pressuring the victim “into doing something,” whereas Patchin and Hinduja (2020, p. 31) defined sextortion as “the threatened dissemination of explicit, intimate, or embarrassing images of a sexual nature without consent, usually for the purpose of procuring additional images, sexual acts, money, or something else.”

### Prevalence

Eight out of 24 studies (33.3%) in our review measured the prevalence of sextortion victimization or perpetration. Three of those studies focused on the experiences of children and adolescents (Finkelhor et al., 2022; Gámez-Guadix, Mateos-Pérez et al., 2022; Patchin & Hinduja, 2020), whereas five prevalence studies focused on adults (Henry et al., 2019, 2020; Pacheco et al., 2019; Powell & Henry, 2019; Trendell, 2019). While all eight prevalence studies measured victimization, only five also measured perpetration (Finkelhor et al., 2022; Henry et al., 2020; Gámez-Guadix, Mateos-Pérez et al., 2022; Patchin & Hinduja, 2020; Trendell, 2019). Regarding victimization among youth, prevalence was relatively low, between 2.6% and 5.0%, and perpetration between 0.7% and 3.0%. Rates of sextortion were higher among adults, with a range between 4.0% and 18.7% for victimization, and 1.6% and 8.8% for perpetration (see Table 3). It is important to note that some adult studies did not ask respondents about experiences they had after the age of 16 or 18. As a result, some respondents may have reported on experiences from childhood or adolescence.

**Table 3.** Prevalence of Sextortion Victimization and Perpetration.

Study	Victimization (%)	Perpetration	*N*
Gámez-Guadix, Mateos-Pérez et al. (2022) ^a^	2.6	0.7%	1,820
Finkelhor et al. (2022) ^a^	3.5	Not reported	1,688
Pacheco et al. (2019)	4.0	Not reported	1,001
Patchin and Hinduja (2020) ^a^	5.0	3.0%	5,568
Henry et al. (2019)	8.6	4.9%	4,274
Powell and Henry (2019)	9.6	Not reported	2,956
Trendell (2019)	17.3	1.6%	630
Henry et al. (2020)	18.7	8.8%	6,109

a Indicates the study was conducted with youth.

## Demographic and Relationship Risk Factors

### Gender

While existing research overwhelmingly shows that men and boys are more likely to perpetrate sextortion than women and girls (CCCP, 2022; Gámez-Guadix, Sorrel et al., 2022; Henry et al., 2019, 2020; Patchin & Hinduja, 2020; Thorn, 2017; Wolak et al., 2016), the findings regarding gender and victimization are mixed. In our review, four studies indicated that sextortion victims were more likely to be boys or men (Eaton et al., 2022; Henry et al., 2019, 2020; Patchin & Hinduja, 2020), while two studies found that women or girls were more likely to be victims (Finkelhor et al., 2023; Kopecký, 2017). In contrast, Gámez-Guadix, Mateos-Pérez et al. (2022) found no significant gender differences in sextortion perpetration or victimization, and Pacheco et al. (2019) reported no gender differences in victimization.

### Age

Patchin and Hinduja (2020) found that no specific age group, within their sample of youth aged 12–17, was more likely to report sextortion victimization or perpetration. In studies on adults, however, there was some consensus that younger people were more likely to report victimization. For instance, Eaton et al. (2022) identified the age group of 18 to 29 as the most likely to report victimization, a trend also observed in Henry et al. (2019, 2020) and Pacheco et al. (2019). Little is known about the correlation between age and sextortion perpetration. Henry et al. (2020) found that younger people aged between 16 and 39 were much more likely than older people aged 40 to 64 to report offending behaviors, but it remains unknown whether children and adolescents are more or less likely to perpetrate sextortion compared to adults.

A difficulty in synthesizing data on age as a risk demographic arises because empirical studies either focus on adults or minors, rather than both, making it challenging to determine whether youth are more likely to experience or perpetrate sextortion compared with adults. Thorn’s (2017) study is an exception. They surveyed 2,097 young people aged between 13 and 25, finding that 47.0% of respondents experienced sextortion before they turned 18 and 53.0% after they turned 18. They also found that one-quarter of respondents reported experiences from when they were under 12.

### Race

Of the 24 articles included in this review, only three (12.5%) examined whether race was a risk factor for either sextortion victimization or perpetration. These studies found that minoritized groups, including indigenous, Black, culturally and linguistically diverse, and migrant groups, were more likely to report sextortion victimization (Eaton et al., 2022; Henry et al., 2019, 2020). One study on Australian adults (Henry et al., 2019) found that 36.0% of Indigenous respondents had experienced threats of intimate image dissemination compared to 8.0% of non-Indigenous Australians. In contrast, Patchin and Hinduja’s (2020) study on U.S. youth found no significant differences in relation to race for victimization or offending.

### Sexuality

Sexual orientation as a risk factor for sextortion was reported in five (20.8%) of the 24 studies included in the review. Findings across these studies show that sexual minorities were more likely to experience sextortion victimization than their heterosexual counterparts (Eaton et al., 2022; Gámez-Guadix & Incera, 2021; Henry et al., 2019; Pacheco et al., 2019; Patchin & Hinduja, 2020). For instance, one study (Gámez-Guadix & Incera, 2021) found that 9.0% of sexual minority adolescents experienced sextortion compared to 3.4% of heterosexual respondents. Only two studies examined sexuality as a risk factor for perpetration, finding that lesbian, gay, bisexual, and nonheterosexual individuals were more likely to admit to sextortion offending (Henry et al., 2020; Patchin & Hinduja, 2020).

### Victim-Perpetrator Relationship

Several studies investigated correlations between other risk characteristics, including the relationship between the victim and the perpetrator. Most of the quantitative studies found that sextortion perpetrators were more likely to be intimate partners or other known persons as opposed to strangers or those known only online to the victim (Finkelhor et al., 2023; Henry et al., 2019; Patchin & Hinduja, 2020; Wolak et al., 2016, 2018). For instance, in Wolak et al.’s (2018) survey of 18 to 25-year-old sextortion victims (*n* = 1,628), 59.0% of respondents knew the perpetrator in person, whereas the remaining 41.0% had only met the person online. Of the in-person relationships, 59.0% were current or former intimate partners, 21.0% were friends or acquaintances, and 15.0% were from a work or school context. In a previous study employing a similar methodology, Wolak et al. (2016) found that most respondents (85.0%) knew, or believed they knew, the perpetrator either “very” or “somewhat” well, of which 64.0% said the perpetrator was a current or former intimate or sexual partner.

In their study on youth, Patchin and Hinduja (2020) found that boyfriends or girlfriends were the most likely group to have perpetrated sextortion (31.7% boys; 32.2% girls), followed by offline “in real-life” friends (26.1% boys; 16.5% girls), online friends (19.9% boys; 11.3% girls), relatives (11.8% boys; 10.4% girls), and someone online who was not well known (4.3% boys; 6.1% girls). In Finkelhor et al.’s (2022) study of youth, they found that adults constituted a minority of offenders (12.0%) and that intimate partners who were a similar age to the victim comprised the largest known group of offenders (31.0%). These findings contrast with those in Thorn’s (2017) study, which found that respondents aged 18 to 25 were nearly equally as likely to have met the perpetrator online compared to an existing offline relationship. In contrast, Eaton et al. (2022) found that strangers were more likely to be perpetrators rather than intimate partners, friends, and online acquaintances. However, it should be noted that in both Thorn (2017) and Eaton et al. (2022), respondents self-selected to participate in the study, which may explain the discrepancy.

### Victim-Offender and Other Experiences Overlap

Finally, several studies noted a correlation between sextortion victimization and perpetration. Patchin and Hinduja (2020) found that half of the youth respondents who reported victimization also admitted to sextortion perpetration. Similarly, they observed that of those who admitted to sextortion offenses, two-thirds also acknowledged being victims themselves. Gámez-Guadix, Mateos-Pérez et al. (2022) and Henry et al. (2019) also found a correlation between perpetration and victimization. In a separate study, Gámez-Guadix, Sorrel et al. (2022) highlighted a strong correlation, particularly among males, between victimization and perpetration in the context of sextortion.

Furthermore, some studies uncovered a high correlation between sextortion and other victimization experiences, such as the nonconsensual sharing of intimate images or sexting (Gámez-Guadix, Mateos-Pérez et al., 2022; Gámez-Guadix & Incera, 2021; Powell & Henry, 2019; Tamarit et al., 2021), digital dating abuse (Wolak et al., 2018), body dissatisfaction (Tamarit et al., 2021), and harassment, stalking, and impersonation (Patchin & Hinduja, 2020).

### Types of Sextortion Offending

The extant research shows that demands, such as in-person sexual favors, explicit images, monetary payments, or having “leverage” over the victim, varied according to context, perpetrator-victim relationship, as well as the gender and age of the victim. For instance, in the Thorn (2017) study, male respondents (aged 18+) were more likely than other age or gender groups to be threatened after two weeks of first contact with an online stranger and were most likely to be threatened for money. While some victims did not comply with the demands, for those who did (e.g., by sending money or further intimate images), this often led to an escalation of threats for more images, money, compliance, or in some instances, sexual intercourse (Thorn, 2017). This finding was also observed in the study by Cross et al. (2022), which examined 258 romance fraud reports made by individual complainants to Australia’s Scamwatch. They found that for men, communications quickly moved to a platform where intimate conversations could take place, including the exchange of intimate images which were then used to immediately blackmail the male victim (in most cases for money). For women, the scenario was more likely to be an established relationship where intimate images had been previously created and shared consensually. In the Wolak et al. (2016) study, the most common demand was for sexual images (51.0%), followed by a demand to stay in, or return to, the relationship with the offender (42.0%). Less common reasons were to meet the victim for either offline (26.0%) or online sexual activity (24.0%), or to obtain money through blackmail (9.0%).

Several studies, including Cross et al. (2022), observed that the use of intimate images tended to function as a tool of escalation, especially when the victim refused to pay the money. The Canadian Center for Child Protection (CCCP, 2022), in their qualitative analysis of the Reddit forum r/Sextortion, also identified escalation as a theme. In nearly all instances (93.0%), when victims sent money, extorters subsequently demanded additional funds. However, the dynamics of sextortion can vary based on the emotional attachment between the victim and the perpetrator. Walsh and Tener (2022) found that in cases with a close emotional attachment, a significant “turning point” often occurred when the victim initiated the separation, or the perpetrator used intimate images to gain more leverage, preventing the end of the relationship.

Only a few studies, both qualitative and quantitative, asked participants whether the threats were actually carried out. In their online survey of young people aged 18 to 25, Wolak et al. (2018) found that in approximately half of the incidents, the threats were carried out, and the images were shared. In Walsh and Tener’s (2022, p. 455) qualitative study, the threat was carried out in 40.0% of cases, with a “variation in the frequency and duration of threats: from a one-time threat to years-long threats.” Finally, in Patchin and Hinduja’s (2020) study, 24.8% of boys and 26.1% of girls experienced the offender posting their images online.

Researchers have also identified the common arenas where sextortion is perpetrated. As highlighted by Walsh and Tener (2022), participants described meeting the offender on Skype, MySpace, Messenger, Facebook, Snapchat, Whisper, Teenspot, Meet Me, and Grindr. In the Thorn (2017) study, they found that 45.0% of respondents reported contact with the perpetrator on multiple platforms, with the most common being social media (54.0%), messaging platforms (41.0%), and video conferencing platforms (23.0%), including Kik, Facebook, and Snapchat (similar findings can be found in Wolak et al., 2016).

In their study, Paquet-Clouston et al. (2019) highlighted the evolving tactics of spammers, such as using bulk emails and demanding cryptocurrency like Bitcoin. Specifically, their examination of sextortion spam campaigns and their financial outcomes revealed that spammers amassed earnings between $1,300,620 and $1,352,266 (USD) in Bitcoin over an 11-month period, averaging a monthly revenue of $122,933 (USD). Additionally, studies have pointed out varying circumstances around victims’ intimate images. Some victims willingly (at least at the time) shared their images with the perpetrator. For instance, Wolak et al. (2016) reported that 71.0% of victims shared explicit images due to an existing intimate relationship. Similarly, in Walsh and Tener’s (2022) study involving 48 participants, 72.0% shared intimate images with the perpetrator. Only a minority of cases involved perpetrators obtaining images without the victim’s awareness, such as through webcam access (Cross et al., 2022; Mandau, 2021).

### Impacts and Harms

Sextortion can result in significant harms, inducing or exacerbating a range of negative emotions, including fear, anxiety, anger, humiliation, shame, and self-blame (Henry et al., 2023; Mandau, 2021; Trendell, 2019; Walsh & Tener, 2022). In some cases, sextortion has led to self-harm, including suicidal ideation and suicide (Walsh & Tener, 2022; Wolak et al., 2018). Gámez-Guadix, Mateos-Pérez et al. (2022) found that victimization was related to symptoms of depression and anxiety compared to perpetration, although noted that more research is needed to establish whether poor mental health outcomes preceded the sextortion experience. Participants in Walsh and Tener’s (2022, p. 457) study described experiencing “extremely negative emotional consequences” as a result of sextortion, including feeling “‘anxious’, ‘frightened’, ‘terrified’, ‘humiliated’, ‘angry’, ‘awkward’, and ‘dirty’.” Participants described a “significant life event” that changed them, and which also included “being constantly nervous, having panic attacks, not being able to eat, experiencing high depression levels, suicide attempts and self-injury” (Walsh & Tener, 2022, p. 459).

Finally, victims of sextortion also report restricting their use of computers, removing themselves from online or offline environments, or closing down their online accounts (Thorn, 2017; Walsh & Tener, 2022). Patchin and Hinduja (2020) documented further harms, such as stalking and harassment (particularly for girls), as well as repeated online or phone contact and impersonation. In Wolak et al.’s (2018) study, approximately one-third of respondents described being threatened with physical assault. Other victims experienced loss of relationships with friends and family (46.0%), left or changed schools, or had school-related issues (14.0%).

### Reporting and Help-Seeking

The third research question addressed in this study pertains to reporting and help-seeking for sextortion; specifically, whether victims reported their experiences, whether they disclosed to others, whether they sought help during or after their experience, what their experiences of reporting and/or help-seeking were like, and what the barriers to reporting or help-seeking were. Reporting refers to making a formal or informal complaint to the police, an online safety agency, digital platform, workplace, school, university, or other institution. Help-seeking refers to obtaining information, advice, and/or support either informally (e.g., from friends, family), or formally (e.g., from psychologists). The research was mixed regarding people’s experiences of both reporting and help-seeking. First, very few studies investigated reporting or help-seeking behaviors. Second, among the studies that did most were quantitative and focused on broader topics like IBSA or technology-facilitated abuse; therefore, their findings on reporting or help-seeking were not necessarily specific to sextortion.

Across the empirical studies on sextortion, most found that police reporting was low (Wolak et al. (2018): 13.0%; Thorn (2017): 17.0%; and Walsh & Tener (2022): 18.0%). Patchin and Hinduja (2020) found that girls were more likely than boys to report to the police. Thorn (2017) found that male victims generally reported a more positive experience (37.0%) with the police compared to female victims (29.0%). Studies also reported low rates of reporting to websites and apps (Patchin & Hinduja (2020): 5.0% (boys) and 7.0% (girls); Wolak et al. (2018): 18.0%; and Walsh & Tener (2022): 45.0%).

Thorn (2017) found that a significant proportion of respondents (one in three) opted to keep silent about their experiences, driven often by feelings of shame, embarrassment, fear of consequences, or the belief that they could manage the situation independently. Notably, around half of the participants chose to confide in a family member or friend, which emerged as a more prevalent disclosure pattern compared to reporting to a platform or to law enforcement (Thorn, 2017). This aligns with findings from Wolak et al. (2018), who observed a similar trend, with nondisclosers expressing feelings of embarrassment or shame (81.0%) and concerns about potential repercussions if they were to share their experiences (68.0%).

In Walsh and Tener’s (2022) study, the majority of the 48 participants shared their experiences with someone, such as a friend, family member, the police, or a therapist. However, the responses they received varied, ranging from helpful and supportive to ambivalent and harmful. They concluded that participants found the responses helpful when recipients actively listened, encouraged them to continue sharing, and showed empathy without judgment or accusation (Walsh & Tener, 2022). Conversely, harmful or unhelpful responses were those that contained judgment, disregard, or a lack of help. Such responses made participants feel frustrated, sad, and lonelier than before disclosing their experiences.

Patchin and Hinduja (2020) noted that, in addition to experiencing a general lack of trust or faith in adults and professionals, adolescents also harbor fears of retaliation, grapple with shame, try to keep their experiences secret, downplay the incidents, lack clarity on who can support them, and often feel lost about where to seek help. They found that almost half of those targeted by sextortion experienced discomfort or felt unable to confide in family or friends about the incident. This was due to feelings of shame, embarrassment, fear of reprisal, or a belief that disclosure would yield no positive outcome. Similarly, in relation to adult women’s experiences, Vitis (2020, p. 36) notes that a lack of family support, combined with the concern about family finding out, “shapes the coercive power of threats.” In other words, the lack of support (or concerns about being blamed and shamed) can increase the likelihood that a victim will feel pressured to comply with the demands.

Among those who did choose to disclose their experiences, Patchin and Hinduja (2020) found that more than half confided in either a friend (55.0% in a friend over 18 and 35.0% in a friend under 18), a parent (37.0%), or an adult family member who was not a parent (17.0%). Only 21.0% reported the situation to the relevant website or app, and only 16.0% reached out to the police. Notably, one in three victims chose not to disclose their sextortion experience to anyone. Many who engaged with law enforcement reported negative responses, including victim-blaming or dismissive remarks (Patchin & Hinduja, 2020; Wolak et al., 2018).

## Discussion

In 2016, a Brookings Institution report noted that the issue of sextortion was “a new sex crime of the digital age” that was “almost entirely unstudied” (Wittes et al., 2016, p. 3). While research on sextortion has expanded in recent years, this review demonstrates that sextortion has not received adequate attention as a standalone phenomenon (see Table 4 for critical findings). The majority of quantitative studies have investigated sextortion alongside other forms of IBSA or online abuse. These surveys included single-item questions or contained very few questions overall on sextortion, given that the focus was on a range of different online harms. Overall, there were only three survey studies out of a total of 16 that exclusively focused on sextortion. Two surveys were conducted with young people aged 18 to 25 (with most reporting on their experiences under the age of 18) (Wolak et al., 2016, 2018), and one was conducted with adults (Eaton et al., 2022). In relation to the study on adults, this was narrowly focused on those who were in a relationship before COVID-19, measuring sextortion experiences during the COVID-19 pandemic (Eaton et al., 2022). None of the three studies provide prevalence estimates, and all were conducted in the United States. There have also been few qualitative studies specifically on sextortion, with only one study conducting interviews with victim-survivors to understand their lived experiences (Walsh & Tener, 2022). More quantitative and qualitative research on sextortion is sorely needed to understand the complexity of this phenomenon and to effectively design interventions to prevent it from happening in the first place.

**Table 4.** Critical Findings.

Terminology	There is no standardized definition of sextortion which hinders comparability. Consensus exists on three defining elements: threat issuance, potential sharing of intimate images, and accompanying demands.
Prevalence	Among adults, victimization ranged from 4.0% to 18.7%. Rates were lower among youth, ranging from 2.6% to 5.0%. Considerable variability in reported prevalence among studies can be attributed to differences in methodologies, sample populations, and definitions of sextortion.
Demographic risk factors	Perpetrators of sextortion are more likely to be men and boys, young people, sexual minorities, or someone known to the victim (e.g., a peer, intimate partner, or former partner). While findings on gender and victimization are mixed, the research indicates that young people and sexual minorities are more likely to experience victimization.
Nature and scope of offending	Sextortion involves a range of demands, including in-person favors, intimate images, and monetary payments. The nature of these demands is influenced by contextual factors, relationship dynamics between individuals, and gender-related considerations. Intimate images are often used as a tool for escalation.
Impacts and harms	Victims of sextortion may experience profound harms, such as fear, anxiety, depression, shame, self-blame, and self-harm. Some studies have found a correlation between sextortion and other adverse experiences, including stalking, harassment, physical violence, and other forms of image-based sexual abuse. However, dedicated studies on the long-term impacts are lacking.
Reporting and help-seeking	Reporting sextortion to the police or digital platforms presents significant challenges. Individuals often face mixed reactions when disclosing to friends, family, or professionals, sometimes encountering negative responses or victim-blaming. Reluctance to disclose is driven by shame and distrust of adults and professionals.

Our review highlighted converging and diverging conceptualizations of sextortion. On a broad level, there was some consensus across the studies about the three core elements that define sextortion: (a) the issuance of a threat; (b) the threat includes the potential sharing of a victim’s intimate (nude or sexual) material (e.g., photos, videos); and (c) the threat is accompanied by a demand imposed on the victim by the perpetrator (e.g., for money, more images, or other unwanted acts). Currently, however, there is a lack of a consistent and clear definition of sextortion as a distinct form of IBSA, as other researchers have also pointed out (Nilsson et al., 2019; Patchin & Hinduja, 2020; Wittes et al., 2016). Notably, some studies in our review did not provide an explicit definition; some defined sextortion broadly as threats to share intimate images, while others specified the demand element. For instance, we found that some studies considered only sexual favors or money as the demand element, while others included a combination of sexual acts, money *and* additional intimate images as potential sextortion demands. We also found that while most studies focused only on threats to share *intimate images*, two studies defined sextortion more broadly as also including threats to share *other intimate content or information* (e.g., evidence of someone visiting a pornographic website) (Buil-Gil & Saldaña-Taboada, 2022; Gámez-Guadix & Incera, 2021).

The inconsistencies in conceptualization create three key limitations. First, the lack of conceptual consistency makes it difficult to compare and synthesize findings across different studies. Second, without a standardized definition, the boundaries between sextortion and other forms of IBSA or technology-facilitated abuse remain blurred. And third, inconsistent or confusing definitions make it difficult for not only researchers but also lawmakers, policymakers, and other practitioners to design and implement suitable interventions.

Overall, our review found that the existing research on sextortion has yielded mixed findings regarding the correlation between certain demographic characteristics and sextortion. Very few studies examined race/ethnicity and/or sexuality. Among those studies that did, there was consensus that victims were more likely to be from minoritized groups. None of the studies examined disability as a risk factor.

Few studies measured prevalence among different age groups: three explored children and adolescents (Gámez-Guadix, Mateos-Pérez et al., 2022; Finkelhor et al., 2022; Patchin & Hinduja, 2020), and five looked at adult populations (Henry et al., 2019, 2020; Pacheco et al., 2019; Powell & Henry, 2019; Trendell, 2019). Victimization rates among adults range from 4.0% to 18.7%, while for youth, the rates are lower, ranging from 0.7% to 5.0%. Similarly, self-reported perpetration prevalence varies, with rates of 0.7% to 3.0% for youth and 1.6% to 8.8% among adults. These results suggest adults experience sextortion at higher rates than children and adolescents, possibly due to greater access and exposure to online spaces where perpetrators operate, as well as perceived financial independence, making adults more lucrative targets for financially motivated offending. Studies with adults suggest that younger adults under the age of 30 are more likely to report victimization and perpetration (although it should be noted that only one study examined age as a risk factor for perpetration among adults) (Trendell, 2019). These results are unsurprising given findings from other research that young people are more likely to fall victim to online scams due to risk tolerance and over-trust in digital platforms and people (Australian Competition and Consumer Commission [ACCC], 2023).

The findings on gender are somewhat mixed, which is consistent with other research on IBSA (Henry et al., 2020). There was strong consensus among the various studies we reviewed that men and boys are more likely to perpetrate sextortion compared to women and girls. One potential explanation for this gendered pattern may lie in the retaliatory dynamics present in male-dominated online spaces, such as gaming communities, where hostile, aggressive, or banter-type/humorous interactions tend to be normalized (Gámez-Guadix, Sorrel et al., 2022). Another explanation is that sextortion is a common tactic in domestic and family violence contexts, where men are more likely to be perpetrators (Henry et al., 2020). Men and boys are also more likely to perpetrate sexual harms more broadly due to sociocultural norms and values, particularly around masculinity and sexuality.

There were also mixed findings in relation to gender and victimization. Most studies found higher victimization rates among boys and men, although two studies found higher rates among women and girls. One possible reason for higher rates of victimization among men could be due to emerging advancements in criminal tactics as well as societal shifts. Recent research suggests transnational organized crime groups may deliberately target men and boys (O’Malley & Holt, 2022). These operations often lure men into online sexual encounters, secretly record the activity, and subsequently threaten exposure unless monetary ransoms are paid (Edwards & Hollely, 2023). Email phishing scams, where perpetrators falsely claim possession of intimate images or evidence of visiting pornographic websites to demand payment, could also disproportionately impact male victims (O’Malley & Holt, 2022). Moreover, perpetrators may adopt deceptive online identities to initiate romantic relationships, later exploiting the emotional connection for financial gain once trust is established (Cross et al., 2023; Edwards & Hollely, 2023; Whitty & Buchanan, 2012). It is possible that men and boys might be more likely to fall victim to sextortion scams when the perpetrator pretends to be a girl or woman and shares an intimate image to prompt the victim to do the same. However, further research is needed to understand the different contexts in which men and boys are more or less likely than women, girls, and nonbinary people to experience sextortion. For instance, more recent research (not included in this review) suggests that women who use video chatting services, messaging applications, and social media platforms are more likely to be victimized by sextortion compared to men (Tavakoli et al., 2024). This further underscores the need to understand the correlation between gender and sextortion victimization and perpetration using mixed methods approaches that can provide a rich understanding of the complexity of this phenomenon.

Our review highlighted variations in the types of sextortion experienced by victims; for instance, perpetrators often use multiple methods, such as social media and mobile text messaging, to demand sexual favors, intimate images, and/or monetary payment. In some instances, demands were less specific and subtle or were more behavioral, such as having leverage or control over the victim. How the sextortion played out depends on age, gender, and the relationship between the offender and victim (which also shapes whether the victim had shared the images with the perpetrator). What the research consistently shows is that intimate images can function as a tool of escalation when the victim fails to comply or does not comply with the demands (e.g., the financial payment is refused or after payment is received). Few studies, though, have examined the consequences or long-term impacts, including whether the intimate images were actually shared.

Another key finding among the quantitative research is that sextortion perpetrators are more likely to be intimate partners or other known persons as opposed to strangers or those known only online to the victim. This is consistent with research on IBSA more broadly (e.g., Henry et al., 2020; Ruvalcaba & Eaton, 2020), as well as research on online child sexual exploitation (Sutton & Finkelhor, 2023). This challenges popular “stranger danger” conceptions of sextortion involving financial scams perpetrated by unknown strangers. What these findings demonstrate is that sextortion is varied in terms of the perpetrator identity, methods, and motivations. Prevention interventions need to be tailored to account for sextortion perpetrated by friends, family members, and intimate partners. In particular, further research is needed on sextortion as part of a broader pattern of abuse within the context of intimate relationships.

Our findings also revealed a strong victim–offender overlap, whereby individuals who report perpetration behaviors also report being a victim of sextortion. However, it is unclear whether there is a causal link between victimization and perpetration. The victim-offender overlap finding is consistent with the findings of research on other forms of IBSA (e.g., Henry et al., 2020). As noted by, this victim–offender overlap might be due to the prevalence of reciprocal online abuse or “banter” on certain sites, apps, or servers, such as online gaming or forums. Moreover, reciprocal sexting might further explain this overlap—as noted by Sparks et al. (2023) in relation to the nonconsensual sharing of intimate images—as romantic partners come into possession of intimate images of one another, when the relationship disintegrates, one partner might threaten to share the other’s intimate images, and they may retaliate in kind and threaten to do the same.

There were significant impacts and harms of sextortion victimization, with victims consistently reporting feelings of fear, anxiety, anger, humiliation, shame, and self-blame, and in some cases, engaging in self-harming behaviors or experiencing other physical effects. Sextortion was found to be reported as a profoundly disruptive life event with significant consequences. Across the research, coping mechanisms also varied, with victims often resorting to restricting their computer use, withdrawing from online or offline environments, closing online accounts, and experiencing changes in schools or relationships.

Finally, in terms of reporting sextortion, our review highlighted that victims of sextortion often face significant challenges and obstacles when attempting to seek help or report their experiences. The persistently low rates of reporting points to a reluctance among victims to engage with law enforcement or digital platforms. Barriers to reporting include feelings of shame, fears of reprisal, and the perception that the situation could be handled independently without involving the police or platforms. The literature consistently points to negative experiences with law enforcement, signaling a critical need for improved training and sensitivity within this sector. Reluctance to disclose is widespread, stemming from feelings of shame and a general lack of trust in adults and professionals. Victims tend to seek solace in informal support networks, with responses varying from helpful to harmful.

### Limitations

This scoping review aimed to synthesize the current literature on sextortion to uncover research gaps, specifically examining what is known about the prevalence of sextortion and demographic risk characteristics, the impacts and harms of sextortion, and the barriers to reporting and/or disclosing experiences. However, several limitations merit attention. First, our focus on literature published exclusively in English may introduce bias by potentially overlooking insights from articles published in other languages. Second, the exclusion of research related to bystanders and societal attitudes in the context of sextortion may also compromise the richness of our understandings. Third, our eligibility criteria were limited to empirical studies focusing on the experiences of victim-survivors or perpetrators of sextortion (e.g., surveys, interviews, reports, online analysis), excluding theoretical articles, legal or media analyses, case studies, as well as studies reporting on interviews with key stakeholders. This additional literature provides rich insights into the phenomenon of sextortion and should be considered in addition to the empirical research. Finally, our review revealed considerable variability in the reported prevalence estimates of sextortion victimization, likely due to differences in study methodologies, sample populations, and definitions of sextortion. Moreover, as mentioned above, most studies did not exclusively focus on sextortion but rather explored it as a subset of IBSA or online abuse more broadly. Given the limited data available, establishing definitive conclusions remains challenging, underscoring the need for more targeted research on sextortion. There was, however, some agreement about demographic risk characteristics, victim impacts, and obstacles to reporting and seeking help.

### Implications for Research, Policy, and Practice

The findings from this review have important implications for research, policy, and practice (see Table 5). First, regarding the definition of sextortion, our findings indicate the need for a clear and standardized definition. Most studies focused on threats to share nude or sexual images and did not include threats to share other content, such as audio recordings, text messages, or other content (e.g., keystroke logging). We suggest that further discussion is needed among a diverse group of stakeholders, including academic researchers, online safety experts, policymakers, and legal practitioners, to determine the parameters of sextortion. This will help to shape further research as well as law, policy, and practice responses.

**Table 5.** Recommendations for Research, Policy, and Practice.

• A clear, standardized definition of sextortion and consideration of whether it should also include threats to share nude or sexual content and information beyond intimate images (e.g., audio recordings, text messages, and other content). • Comprehensive quantitative survey research on sextortion among children, adolescents, and adults should investigate prevalence, demographic risk characteristics, victim–perpetrator relationships, bystander attitudes and interventions, impacts, help-seeking, and reporting. • In-depth qualitative research involving both victim-survivors and perpetrators should explore lived experiences and individual and structural drivers of sextortion, including perpetrator motivations, impacts of victimization, and barriers to reporting and seeking help. • Longitudinal research to examine the long-term effects of sextortion on victims. • An intersectional approach to examine how social identities intersect and shape experiences of sextortion, as well as help-seeking and reporting. • Targeted and nonjudgmental awareness campaigns, cybersecurity initiatives, and respectful relationships education to address problematic gender norms and behaviors, and educate people about risks and consequences of sextortion, and provide guidance on how to seek help and report incidents. • Evaluation of educational programs. • Specialized training and resources for law enforcement agencies, teachers, parents, victim–support services, and other organizations to better detect, prevent, and respond to sextortion. These should be culturally sensitive, victim-centered, and trauma-informed. • More resources for information, support, and advice for victim-survivors as well as potential perpetrators, including online reporting options, dedicated helplines, chatbots, apps, and other digital tools. • Proactive tools designed with safety-by-design and privacy-by-design principles that can be used to automatically detect sextortion on digital platforms (e.g., algorithms, AI detection tools); respond to user reports of sextortion in a timely and sensitive manner with consequences for perpetrators (e.g., content removal; account suspension); and prevent sextortion (e.g., digital hashing, nudges, warnings, educational information, safer default account settings). • Greater collaboration among various organizations, especially technology companies, law enforcement agencies, online safety organizations, and schools. • Implementation of specific and consistent criminal and civil statutes addressing sextortion, along with increased legal resources to raise awareness and support for victim-survivors.

Second, further qualitative and quantitative research is sorely needed to provide a comprehensive understanding of sextortion victimization and perpetration. The growth of sextortion as a form of IBSA has been rapid, and there is an urgent need for comprehensive, targeted research to explore not only the prevalence, patterns, and risk factors associated with sextortion but also the lived experiences of victim-survivors, including the obstacles they face in help-seeking and reporting. Future research should explore the correlation between sextortion and other victimization experiences, such as intimate partner abuse. Future research should also encompass a broader range of demographic factors, including race/ethnicity, sexuality, age, gender, and disability. This research should examine how these categories intersect to shape experiences. In particular, there is a significant gap in sextortion research on the experiences of nonbinary, genderqueer, and transgender individuals. These populations may face unique vulnerabilities and experiences that differ from those of cisgender individuals, highlighting the need for further research in this area.

Third, in terms of prevention, educational initiatives, including awareness campaigns, cyber security, digital literacy, and respectful relationships education, should focus on addressing problematic gendered norms and behaviors, as well as educating people about the methods, risks, and consequences of sextortion, and strategies and channels for reporting and help-seeking. It is vital that education on sextortion proactively addresses the stigma related to sextortion through careful attention to language to avoid victim-blaming—this not only significantly hampers reporting and help-seeking but can contribute to depression, anxiety, and the propensity for self-harm. Prevention, however, should not be solely the responsibility of educational institutions. Technology companies also need to continue investing in innovative prevention tools and features, such as digital hashing (e.g., the “Take It Down” and “Stop NCII” tools), “nudges” to warn people about sharing intimate images, nudity protection, and blurring tools to protect people from seeing content, educational messaging, and safer default account settings, among a range of other measures.

Fourth, training, education, and collaborative approaches are imperative for law enforcement, teachers, parents, victim-support services, and others to be able to detect, prevent, and respond to sextortion. Specialized training on sextortion, including tactics used by perpetrators, the impacts and harms on victims, and the legal frameworks provisioning sextortion, can equip people with the knowledge and skills to identify and investigate sextortion, as well as support victims throughout the process. These initiatives must adopt victim-centered approaches, promoting empathy and challenging victim-blaming attitudes. Ultimately, an increased understanding of sextortion can assist in creating safe and supportive environments for victim-survivors to disclose their experiences, while respecting their autonomy and agency throughout the investigation process, as well as improving reporting channels and access to support services.

Fifth, more resources are needed to support victim-survivors to report their experiences and seek help. These resources need to be culturally appropriate and tailored to accommodate particular at-risk and vulnerable populations. Resources are needed to help victim-support services provide timely, sensitive, and trauma-informed support. Resources are also needed to empower victim-survivors, including online reporting tools (e.g., to police or digital platforms), dedicated helplines and hotlines, chatbots, and mobile applications designed specifically to provide information and access to reporting and help-seeking options. Technology companies also need to implement safety-by-design and privacy-by-design principles in the design of new products and proactively develop tools and algorithms for detecting and responding to sextortion on their platforms.

Finally, in relation to legal responses, many jurisdictions internationally lack specific legislation on sextortion and rely on existing laws, such as blackmail, which are often unsuitable for addressing this crime. The absence of specific and consistent sextortion legislation has arguably hampered reporting and prosecution. Although there are significant limitations associated with legal responses to sextortion, including identifying the perpetrator, collecting forensic evidence, working across borders and legal jurisdictions, and the sometimes poor treatment of victim-survivors in the legal system, introducing specific criminal and civil penalties for sextortion remains an important step in communicating the seriousness of these behaviors, ensuring effective prosecutions, and allocating legal and other resources. Introducing specific penalties for sextortion through law can also further justify prevention and response interventions, as well as compel technology companies into action.

## Conclusion

This scoping review aimed to synthesize the existing scholarly literature on sextortion. Our results indicate that despite increasing media attention on sextortion, empirical research remains scarce. The research shows that sextortion is multifaceted, encompassing a range of acts, including grooming, computer hacking, sexting within romantic relationships, sex trafficking, and financial scams like “romance fraud.” Perpetrators range from intimate partners and family members to friends, acquaintances, strangers online, and organized crime groups. Motivations range from financial gain and sexual gratification to power, control, or simply retaliation. Studies reveal significant psychological, physical, social, and economic impacts on victims, including heightened risks of depression, anxiety, suicidal thoughts, and, in extreme cases, suicide.

In conclusion, our review provides a comprehensive overview of terminological variations, prevalence rates across age groups, risk factors for victimization, methods of sextortion offenses, resulting harms, and barriers to reporting and seeking support. These findings highlight critical gaps in awareness, understanding, prevention strategies, and evidence-based interventions while laying the groundwork for further research and informed action across education, policymaking, law enforcement, and support services. Comprehensive solutions are urgently needed by various stakeholders, including researchers, policymakers, law enforcement, educators, and support service providers, to reduce the occurrence of sextortion and to address the disproportionate impacts sextortion has on vulnerable populations. These solutions should be paired with efforts to promote safe and accessible channels for reporting sextortion. Developing a stronger understanding of sextortion through further research will improve societal awareness, shape policies, law enforcement, and prevention efforts, and enhance victim support services.

## Notes

1. Image-based sexual abuse (IBSA) refers to the nonconsensual taking, creating, or sharing of intimate images (nude or sexual photos or videos), including threats to share intimate images. This type of abuse can also involve, pressuring, coercing, or threatening someone to share their intimate images, sending unwanted and unsolicited intimate images, and creating digitally altered images, including “deepfakes” (see Henry et al., 2020).

2. Technology-facilitated sexual violence (TFSV) refers to the “diverse ways in which criminal, civil or otherwise harmful sexually aggressive or harassing behaviors are being perpetrated with the aid or use of digital communications technologies” (Powell & Henry, 2017: 5).
